# Highly robust model of transcription regulator activity predicts breast cancer overall survival

**DOI:** 10.1186/s12920-020-0688-z

**Published:** 2020-04-03

**Authors:** Chuanpeng Dong, Jiannan Liu, Steven X. Chen, Tianhan Dong, Guanglong Jiang, Yue Wang, Huanmei Wu, Jill L. Reiter, Yunlong Liu

**Affiliations:** 1 Department of Medical and Molecular Genetics, Center for Computational Biology and Bioinformatics, Indiana University School of Medicine, Indianapolis, IN 46202 USA; 2 Department of BioHealth Informatics, School of Informatics and Computing, Indiana University-Purdue University Indianapolis, Indianapolis, IN 46202 USA; 3 Department of Pharmacology and Toxicology, Indiana University School of Medicine, Indianapolis, IN 46202 USA

**Keywords:** Breast cancer, Transcription regulators, Prognostic model

## Abstract

**Background:**

While several multigene signatures are available for predicting breast cancer prognosis, particularly in early stage disease, effective molecular indicators are needed, especially for triple-negative carcinomas, to improve treatments and predict diagnostic outcomes. The objective of this study was to identify transcriptional regulatory networks to better understand mechanisms giving rise to breast cancer development and to incorporate this information into a model for predicting clinical outcomes.

**Methods:**

Gene expression profiles from 1097 breast cancer patients were retrieved from The Cancer Genome Atlas (TCGA). Breast cancer-specific transcription regulatory information was identified by considering the binding site information from ENCODE and the top co-expressed targets in TCGA using a nonlinear approach. We then used this information to predict breast cancer patient survival outcome.

**Result:**

We built a multiple regulator-based prediction model for breast cancer. This model was validated in more than 5000 breast cancer patients from the Gene Expression Omnibus (GEO) databases. We demonstrated our regulator model was significantly associated with clinical stage and that cell cycle and DNA replication related pathways were significantly enriched in high regulator risk patients.

**Conclusion:**

Our findings demonstrate that transcriptional regulator activities can predict patient survival. This finding provides additional biological insights into the mechanisms of breast cancer progression.

## Background

Breast cancer is the most frequently diagnosed cancer and the second leading cause of cancer deaths in women worldwide [1]. In the United States, breast cancer accounted for 30% of all new cancer cases and 14% of cancer deaths for women in 2017, with an estimated 252,710 newly diagnosed cases and 40,610 deaths [2]. However, because of intertumoral and intratumor heterogeneity, significant challenges exist in designing effective treatment regimens and in predicting clinical outcomes.

Currently, breast tumor classification is primarily based on histopathologic features and the expression of estrogen receptor (ER), progesterone receptor (PR) and human epidermal growth factor receptor 2 (HER2) [3]. These subtypes differ with respect to available receptor-targeted therapies, response to treatment, clinical outcomes and risk of acquiring resistance to therapy [4]. Hundreds of other biomarkers have been reported in breast cancer for prognostic and therapeutic applications [5]. Over the past decade, multi-gene signatures have been developed for breast cancer subtyping and risk stratification [6]. For instance, the PAM50 gene signature measures the expression levels of 50 genes in breast cancer samples to classify a tumor as one of five intrinsic subtypes (luminal A, luminal B, HER2-enriched, basal-like and normal like), and it has prognostic value in both untreated and tamoxifen treated patient populations [7, 8]. The MammaPrint assay categorized patients into good or poor risk groups using 70 genes and has been approved by the Food and Drug Administration (FDA) to aid in predicting prognosis for breast cancer patients with specific clinical characteristics [9, 10]. However, these gene-based risk models have certain limitations and to date, there is no multigene test that has been approved for recommending adjuvant treatment for triple-negative (ER/PR/HER2-negative) breast tumors. There remains a critical need for the development of a robust model that can aid in effectively predicting individual patient prognosis for hormone-receptor-negative breast tumors that can convey additional biological information from gene expression profiles.

Transcriptional regulators, including the transcription factors, cofactors, chromatin remodelers, histone modification proteins and other DNA binding proteins, play fundamental roles in many cellular processes including response to extracellular and intracellular signals. By binding to promoter regions of genes, transcription regulators are able to up- or down-regulate specific target genes, thereby affecting many cellular activities. In recent years, several studies have implemented machine-learning methods in cancer prognostic biomarker development. For example, a support vector machine (SVM)-classifier has been used to construct a breast cancer prognostic signature consisting of 10 microRNAs that accurately predicted breast cancer stage [11]. Moreover, a clustering-based method identified microRNA combinatorial biomarkers with high accuracy and efficiency [12]. However, there are challenges with using machine-learning methods with high-dimensional profiles [13].

We hypothesized that transforming gene expression profiles into transcription regulator activity levels would reduce the dimensionality while retaining the most useful information from the gene expression profile during feature selection and the model training process. Herein, we built a transcription regulator-based model by mining gene expression profiles from 1097 breast cancer patients obtained from The Cancer Genome Atlas (TCGA). We implemented a rank-based score function to estimate the regulator activity levels to build the prediction model. In addition, the predictive power of this transcription regulator activity-based model was validated on over 5000 breast cancer patient profiles in the Gene Expression Omnibus (GEO) database. Gene set enrichment analysis demonstrated that the transcriptional regulator-based prognostic signature identified key pathways involved in breast cancer development and progression. Thus, transcription regulator activity models are expected to provide new information that could be used to develop new treatment strategies for breast cancer.

## Methods

### Gene expression profiles

Breast cancer gene expression datasets and the corresponding clinical data were downloaded from TCGA and GEO databases. TCGA breast cancer RNA-sequencing data (log2(norm_count + 1) normalized) [14], including the corresponding clinical information, were downloaded from UCSC Xena (http://xena.ucsc.edu, version 2017-10-13) [15]. Validation microarray datasets from 29 studies were downloaded from GEO (Additional file 1: Table S1). For the Affymetrix microarray studies, raw data of microarray datasets were downloaded in CEL format or processed soft matrix files were used for datasets without CEL. After background correction, the robust multi-array average (RMA) method was used to normalize the Affymetrix microarray data [16]. For microarray studies using other platforms, the series matrix files were downloaded from the NCBI GEO website [17]. Probes were annotated to the gene symbols, and multiple probes annotated to the same gene were merged and mean values were calculated as the expression of the corresponding genes. The clinical data were acquired from the respective literature citations.

### Chip-seq datasets from ENCODE

The transcriptional regulators and their target genes list was retrieved from the ENCODE project via ChIPbase V2 (http://rna.sysu.edu.cn/chipbase/) [18, 19]. The transcription factor and chromatin remodeling factor regulatory domains were defined using the following settings: 1 kb upstream and downstream of the target gene transcription start sites, union combination mode and all motifs. The resulting lists were then limited to ENCODE as the source. In total, 180 transcription factors or other chromatin remodeling factor data were included and together, are referred to as transcription regulators in this study.

### Survival analysis and statistical methods

The Cox proportional-hazards model was used to select the candidate transcription regulator that significantly associated with patients’ overall survival [20, 21]. A multivariate Cox regression was performed to weight each of the selected potential transcription regulators with an adapted coefficient. A transcriptional regulator activity based risk score formula was constructed by including statistically significant genes weighted by their estimated multivariable Cox’s regression adapted coefficients [22].

$$ \mathrm{Risk}\ \mathrm{score}={\sum}_1^n\  coefficient(i)\ast transcription\ regulator(i)\  activity. $$

Patients were then divided into high-risk or low-risk groups using the median risk score as the cutoff [23]. The Kaplan-Meier curve was used to compare the survival probabilities between the high-risk and low-risk groups, and the log-rank test was adopted to test the difference in survival rate between patients in the high- and low-risk subgroups. A *p*-value less than 0.05 was considered as statistically significant. The survival analyses were conducted with the *survival* package in the statistical environment R (v3.5.1). Statistical computing and visualization were conducted with R.

Gene set enrichment analysis was conducted with GSEA software (http://www.broadinstitute.org/gsea) [24] using the canonical pathways collection (version, c2.cp.v6.2) [25] and a false discovery rate (FDR) value less than 0.01 after performing 1000 permutations was considered to be significant.

## Results

### Dataset and workflow for estimating transcription regulator activities

RNA-seq profiles from 1097 primary breast cancer patients were used as the training set. Transcription regulator binding site information was extracted from ChIP-seq data contained within ENCODE. This data included 180 regulators and their gene targets. Additionally, 29 breast cancer studies in GEO were used as a validation set (Additional file 1: Table S1).

**Fig. 1:**
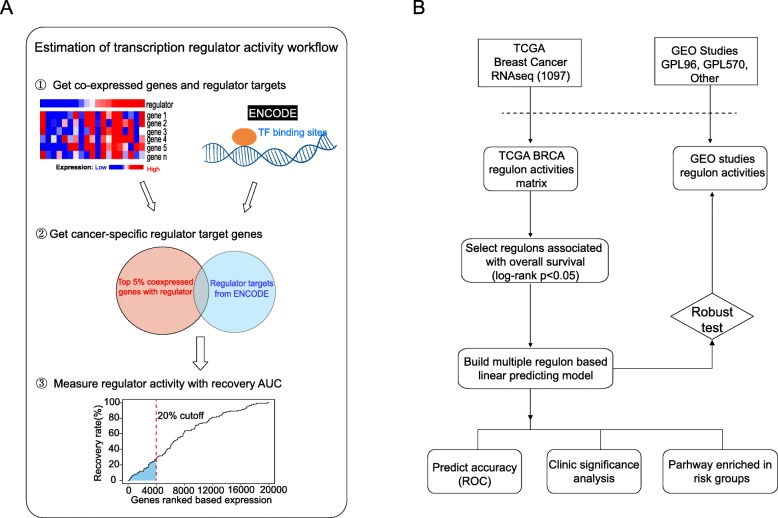
Workflow of the study. **a**. Estimation of transcription regulator activities workflow. Co-expression analysis of each regulator with all genes in TCGA breast cancer cohort was performed using GRNBoost2 and regulator binding site information was retrieved from ENCODE (step 1). The intersection of the top 5% of co-expressed genes with regulator target genes was identified (step 2) and used to calculate the regulator activity with a rank-based approach (step 3). **b**. Overall workflow of the study. Abbreviations: TCGA, The Cancer Genome Atlas; GEO, Gene Expression Omnibus

We estimated transcription regulator activity using a method that was adapted from the single cell algorithm SCENIC [26]. The workflow for obtaining this estimate followed three steps as shown in Fig. 1. Firstly, co-expression analysis for each of the 180 transcription regulators with all genes in the TCGA breast cancer sets was conducted using GRNBoost2, an efficient algorithm for regulatory network inference using gradient boosting. Secondly, the intersection of the top 5% of transcription regulator-gene pairs (i.e, regulons) from GRNBoost2 [27] and genes from the ENCODE ChIP-seq dataset produced the set of genes considered to be the regulator targets that were expressed in breast cancers. Thirdly, the activity of each transcription regulator in each patient was measured with the rank based approach AUCell package [28]. We calculated the enrichment of the identified breast cancer specific regulator target as an area under the recovery curve across the ranking of all genes in a particular patient’s profile. The output is the enrichment matrix of transcription regulator activity for each patient; genes were ranked by their expression value and the cutoff parameter was set as 0.2 for both the training and testing sets. The rank-based method enabled estimation of transcription regulator activity at the individual patient level.

### Transcription regulator activities correlated with breast cancer subtypes

**Fig. 2:**
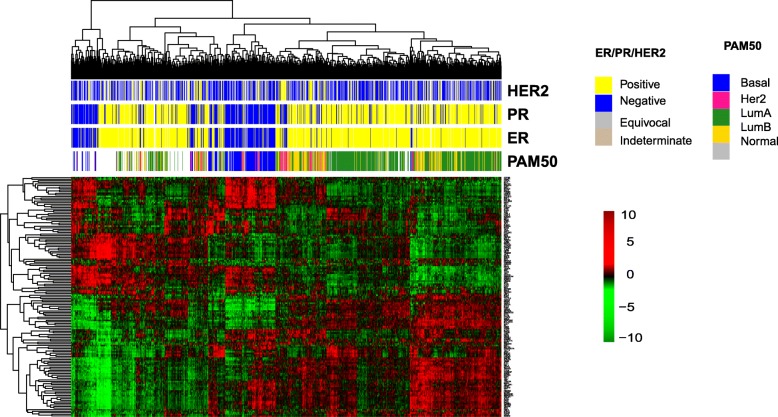
Correlation of transcription regulator activities with breast cancer subtypes. The distribution of transcription regulator activity and breast cancer subtype with hormone receptor status and PAM50 molecular classification. The transcription regulator activity profile was generated by unsupervised cluster analysis, in which the rows represented the regulator activities and the columns represented the samples. The transcription regulator activity score varies between − 10 and + 10 as indicated by a gradient from green to red color

We postulated that transcription regulator activity might be similar in breast cancers with similar gene expression profiles. This would suggest that breast tumors within the same molecular subtypes might have similar transcription regulator profiles. To address this question, we asked whether a correlation existed between commonly used molecular PAM50 subtypes and the transcriptional regulator activity. Each transcriptional regulator activity was calculated by the approach described above. We then conducted an unsupervised clustering with the regulator activity matrix. These results indicated that transcription regulator activity specifically identified the basal-like breast tumors (Fig. 2). Transcriptional regulators that exhibited higher activities in the basal-like tumors compared to the other breast cancer subtypes include BRF1, CTCFL, E2F1, FOXM1, GTF2B, GTF3C2, HCFC1, KAT2A, MEF2C, MYBL2, MYC, POLR3G and WRNIP1. These findings are consistent with a previous report that FOXM1 functions as a specific marker for triple negative breast cancer [29]. In addition, previous studies have demonstrated that triple-negative tumors exhibit elevated expression of MYC regulatory genes and increased activity of the MYC pathway [30].

### Transcriptional regulator activity was associated with breast cancer prognosis

We next asked whether transcription regulator activity would be useful in predicting breast cancer patient prognosis. To address this question, we applied the Cox proportional-hazards model to screen transcription regulators that correlated with the overall survival of breast cancer patients. We found that fifteen transcription regulator activities showed significant associations with overall survival (Table 1). Among those 15 regulator activities, four transcriptional regulators had hazard ratio above 1 (CHD7, REST, TBP and THAP1), indicating that elevated activities of these transcriptional regulators were associated with poor prognosis. Eleven transcription regulators (ATF3, BATF, ESR1, FOXA1, GTF2B, IKZF1, IRF1, JUNB, KDM1A, MAX and STAT2) had a hazard ratio less than 1, suggesting that higher activity of these transcription regulators in breast tumors might be beneficial for patient survival.

**Table 1:** Transcriptional regulator activity associated with breast cancer overall survival

Regulator	log-rank p	HR	95% CI	*p* value
BATF	3.00E-06	0.45	(0.32–0.64)	5.26E-06
ESR1	0.002	0.60	(0.43–0.83)	0.0023
IRF1	0.0034	0.62	(0.44–0.85)	0.0037
THAP1	0.0099	1.53	(1.10–2.12)	0.0105
KDM1A	0.0158	0.67	(0.48–0.93)	0.0165
TBP	0.0167	1.49	(1.07–2.06)	0.0173
JUNB	0.0199	0.68	(0.49–0.94)	0.0206
ATF3	0.021	0.69	(0.50–0.95)	0.0218
CHD7	0.0236	1.45	(1.05–2.01)	0.0243
STAT2	0.032	0.70	(0.51–0.97)	0.0329
GTF2B	0.04	0.71	(0.52–0.99)	0.0409
IKZF1	0.0406	0.71	(0.52–0.99)	0.0416
FOXA1	0.0459	0.72	(0.52–1.00)	0.0469
MAX	0.046	0.72	(0.52–1.00)	0.0469
REST	0.0493	1.38	(1.00–1.91)	0.0502

*HR Hazard ratio, *CI* confidence interval

### Transcriptional regulator model predicted breast cancer overall survival

**Fig. 3:**
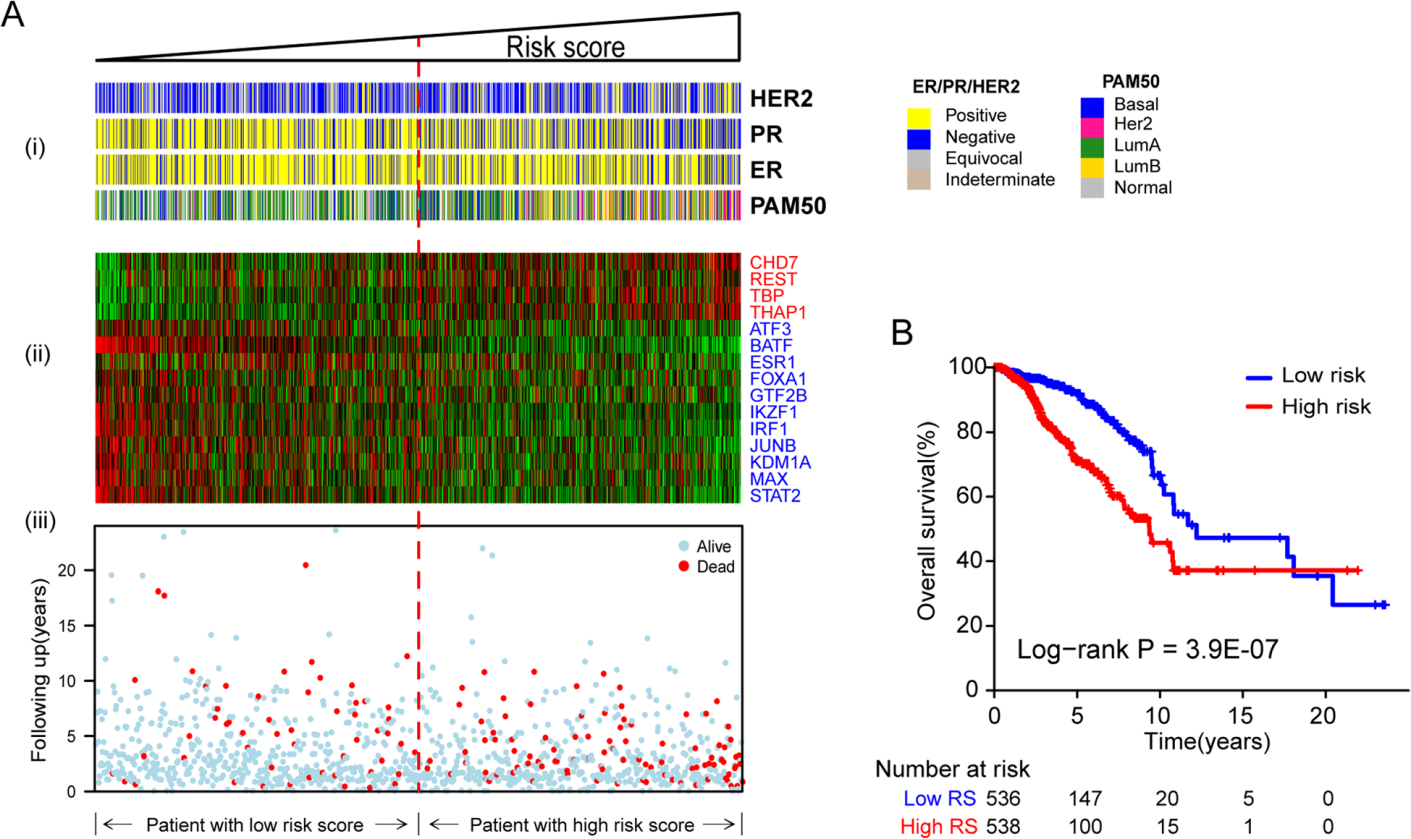
Regulator based risk model of TCGA breast cancer patients. **a** The distribution of the significant regulator activities, patients’ survival status and gene expression signature were analyzed in the TCGA breast cancer patients. (i) Hormone receptor status and PAM50 molecular classification of breast cancer patients. (ii) Heatmap of the selected regulator activities profile. (iii) Patients overall survival status and time. Rows represent genes, and columns represent patients. The red dashed line represents the risk score median cutoff dividing patients into low-risk and high-risk groups. **b** Kaplan-Meier estimates of patient survival in high- and low-risk groups based on transcriptional regulator activities

We then calculated risk scores for each breast cancer patient in the training set as described in the Methods section. Patients were divided into low-risk and high-risk groups using median risk score as the cut-off. The risk scores distribution, survival status and transcription regulator profiles for the TCGA training set are shown in Fig. 3a. Our results show that patients in the high-risk group had significantly shorter overall survival time than those in the low-risk group (log-rank *P* < 0.0001) (Fig. 3b).

In addition, we found that more hormone receptor negative (ER- and PR-negative) breast cancer patients were assigned to the high-risk group. Likewise, the basal-like and HER2-enriched breast cancer patients also were in the high-risk group, which is consistent with the clinical findings [31] that HER2-enriched and lumB subtypes of breast cancer have a poor prognosis compared with the lumA and normal-like breast cancers.

### Validation of the transcription regulator model with independent datasets

**Fig. 4:**
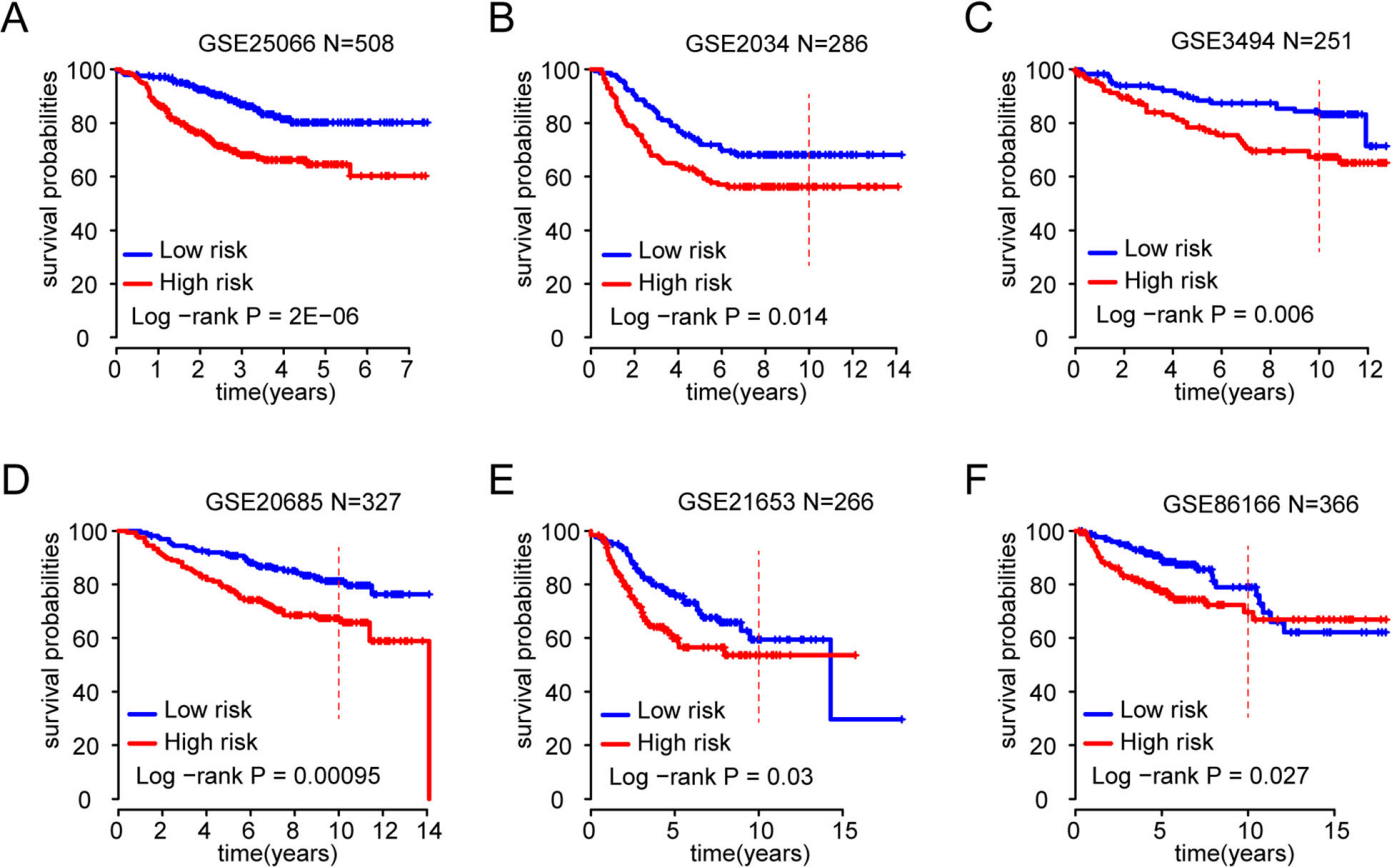
Validation of the transcriptional regulator model with GEO datasets. Kaplan-Meier estimates of the survival of independent breast cancer datasets using the regulator activity signature. **a** Kaplan-Meier curves of overall survival for **a** GSE25066, **b** GSE2034, **c** GSE3494, **d** GSE20685, **e** GSE21653 and **f** GSE86166. The low-risk and high-risk groups of patients was determined on the basis of the median risk score for each validation dataset. The tick marks on the Kaplan-Meier curves represent censored subjects. The statistically significant differences between the two curves were determined by the two-sided log-rank test. The vertical red dashed line indicates the 10-year mark that was used for interpreting survival difference between groups

To test whether the high performance of the risk-score model in the training dataset might have resulted from overfitting, we evaluated the performance of the transcription regulator activity model using independent breast cancer datasets from the GEO. Consistent with the training set, patients with high risk scores showed a clear trend of decreased survival rate in 23 of 29 GEO studies (Fig. 4 a-f and Additional file 1: Figure S1).

Notably, these independent validation GEO sets used different gene-expression platforms, such as the Affymetrix Human Genome U133A/B Array (Fig. 4 a-c), Affymetrix Human Genome U133 Plus 2.0 Array (Fig. 4 d and e) and the Illumina platform (Fig. 4f). These data strongly suggest that patients assigned to the high-risk group had significantly decreased 10-year survival, compared with those patients in the low risk groups. Taken together, we conclude that this risk-score model shows robust performance across datasets and platforms. We also tried to compare our regulator model with an existing genomic classification assay. MammaPrint risk for each patient in the TCGA training set and the GEO validation breast cancer set was measured with the genefu package in R 3.6.0 [32]. The results are shown in Additional file 1: Figure S3. The ROC curve showed that our regulator-based model performed better than the MammaPrint risk model in the two largest testing sets GSE25066 (Additional file 1: Figure S3A) and GSE20685 (Additional file 1: Figure S3B).

### Association of transcription regulator model with clinical and molecular features

**Fig. 5:**
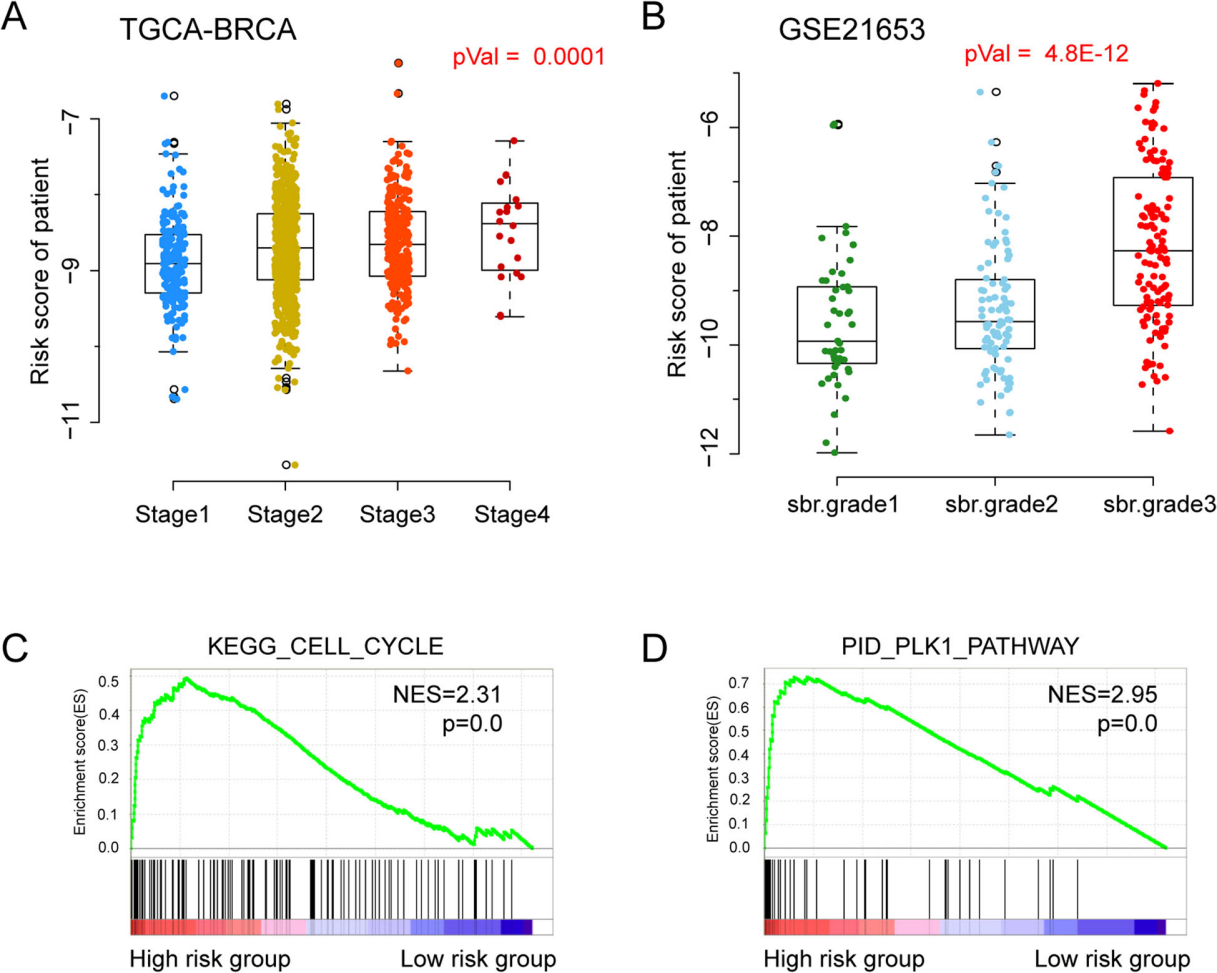
Association of the transcription regulator-based risk score with pathological and molecular features. The regulator risk score is shown for breast cancer stages in the TCGA cohort (**a**) and in a selected validation set GSE21653 (**b**). One-way ANOVA *p* values are provided. Cancer related pathways that were significantly altered in patients with high regulator risk scores included cell cycle (**c**) and PLK1 signaling pathways (**d**). Normalized enrichment score (NES) was used to evaluate the enrichment results

To explore potential molecular mechanisms that might contribute to the clinical association with the transcription regulator activity-based model, we analyzed the correlation between the regulator risk score and the cancer clinical stage. We found the transcription regulator-based risk score and the pathological stage of breast cancer patients was correlated in both the training and validation sets (Fig. 5 a and b), where the one-way ANOVA *p* value reached 0.0001 for TCGA and p value was 4.8E-12 for GSE21653.

In addition, we performed Gene Set Enrichment Analysis (GSEA) on the TCGA breast cancer cohort. Compared with the patients assigned as low-risk, the high-risk patient group showed that the top two enriched pathways were cell cycle (Fig. 5c) and PLK signaling (Fig. 5d), which both play critical roles in cancer initiation and development. These findings provide evidence that the risk score can provide information relevant to potential molecular mechanisms that might be involved in tumor progression and survival outcome in breast cancer patients.

## Discussion

In this study, a transcriptional regulator-based model was established for predicting the prognosis of breast cancer. By transforming gene expression profiles to transcription regulator activity levels, we demonstrated that regulon activity can be used to explore breast cancer gene expression data. After identifying that regulons significantly associated with breast cancer overall survival, we further constructed a multi-regulator activity-based prediction model for breast cancer. The finding from the training set was validated in different independent GEO sets (> 3500 patients), which indicates that the regulator activity-based model is robust.

Among the regulators that were used in the model, several are known to play a role in breast cancer. The chromatin remodeler CHD7 is one of the most commonly amplified CHD genes in breast cancer, and mRNA expression levels of CHD7 are significantly upregulated in basal-like breast cancer [33]. The histone demethylase KDM1A, which functions to repress and activate transcription by mediating histone H3K4me1/2 and H3K9me1/2 demethylation, respectively, was reported to be present at significantly lower levels in breast cancer samples compared with normal tissues [34, 35]. Consistent with the previous literature, we found KDM1A was significantly lower in those patients with worse survival. Silencing of THAP–zinc finger protein THAP1 inhibits endothelial G1/S cell-cycle progression [36]. High FOXA1 was associated with better breast cancer specific survival among ER-positive breast cancer [37]. Functional studies have demonstrated that JUNB plays a pro-survival role in breast cancer cells in response to a lethal dose of flavopiridol [38]. Low mRNA expression of ESR1 is a determinant of tamoxifen resistance in ER-positive breast cancer [39]. The majority of the selected transcription regulators had been demonstrated previously to be strongly associated with breast cancer progression; therefore, differences in crucial regulator activity between low- and high-risk groups may provide new information for better understanding breast tumor pathology and risk for recurrence.

We hypothesized that limiting a prognostic gene signature to transcription regulators would reduce the dimensionality of the expression profile during feature selection and model training process. Additionally, we predicted that such a transcription regulator activity profile would be less susceptible to variability in the expression of individual genes and may thereby improve the prognostic significance of tumor gene profiling. To the best of our knowledge, a breast cancer prognostic signature with high prediction power has not yet been constructed using only information provided by transcription regulatory factors. Compared with other published molecular signatures and panels for breast cancer, this transcriptional regulator-based signature was highly robust across different datasets and platforms with very large-scale breast cancer samples, as the TCGA data was from RNA-sequencing, while various microarray platforms were used in GEO. The robustness of this model arises primarily from two aspects. Firstly, each transcriptional regulator activity was estimated using hundreds of direct target genes by considering both co-expression and ENCODE results, which produced a highly stable model without perturbation due to the variability of single gene expression levels. Secondly, the transcription regulator activity estimation was based on the rank of the absolute expression value of its target genes, which enabled it to perform well across different gene expression platforms. In addition, the transcriptional regulator activities were estimated in a tissue-specific manner, which allows the regulator activity based prognostic risk score to deliver additional biological information from breast cancer mRNA expression profiles.

Limitations of this study include the assumption that mRNA expression levels from the different platforms were measured appropriately and reflect the actual mRNA abundance of each gene. Secondly, the binding targets of the regulators obtained from ENCODE were not specific to breast cancer, so it is possible that a given regulator might not bind to all of the same targets in breast tumors. Thirdly, the cutoff for each dataset was determined separately based on the median risk score of the respective dataset; however, we did find that the value of the risk scores were in a similar range (− 12 to − 5).

## Conclusion

In the present study, we built a robust model for predicting overall survival based on biologically relevant transcriptional regulator information and further validated it using large cohorts of breast cancer patients. The transcription regulator model should enhance our understanding of breast cancer progression and guide personalized treatment selection.

## Supplementary information

**Additional file 1: Table S1.** GEO datasets used in this study. **Figure S1.** Transcriptional regulator model predicts survival in breast cancer datasets. **Figure S2.** The regulators risk groups associated with average survival time. **Figure S3.** Comparison of the performance of the regulator model with MammaPrint.

## Data Availability

The datasets analyzed in the current study are publicly available in TCGA (https://portal.gdc.cancer.gov and https://tcga.xenahubs.net/download/TCGA.BRCA.sampleMap/HiSeqV2.gz). The microarray studies analyzed during the current study, GSE25066(https://www.ncbi.nlm.nih.gov/geo/query/acc.cgi?acc=gse25066), GSE2034(https://www.ncbi.nlm.nih.gov/geo/query/acc.cgi?acc=gse2034), GSE3494(https://www.ncbi.nlm.nih.gov/geo/query/acc.cgi?acc=gse3494), GSE20685(https://www.ncbi.nlm.nih.gov/geo/query/acc.cgi?acc=gse20685), GSE21653(https://www.ncbi.nlm.nih.gov/geo/query/acc.cgi?acc=gse21653) and GSE86166(https://www.ncbi.nlm.nih.gov/geo/query/acc.cgi?acc=gse86166), are available in the GEO Datasets repository.
