# Supplementary material for: Highly robust model of transcription regulator activity predicts breast cancer overall survival
Source: BMC Med Genomics. 2020 Apr 3;13(Suppl 5):49. doi: 10.1186/s12920-020-0688-z (PMC7118819; doi:10.1186/s12920-020-0688-z)
Supplement: Supplementary file 1 — Additional file 1: Table S1. GEO datasets used in this study. Figure S1. Transcriptional regulator model predicts survival in breast cancer datasets. Figure S2. The regulators risk groups associated with average survival time. Figure S3. Comparison of the performance of the regulator model with MammaPrint. [file 12920_2020_688_MOESM1_ESM.docx]

**Additional files**

Supplementary tables and figures

| **Series** | **No. Patient** | **Platform** |  | **Series** | **No. Patient** | **Platform** |
| --- | --- | --- | --- | --- | --- | --- |
| **GSE1456** | 159 | Affymetrix Human Genome U133A Array |  | **GSE9195** | 77 | Affymetrix Human Genome U133 Plus 2.0 Array |
| **GSE2034** | 286 | Affymetrix Human Genome U133A Array |  | **GSE19615** | 115 | Affymetrix Human Genome U133 Plus 2.0 Array |
| **GSE5327** | 58 | Affymetrix Human Genome U133A Array |  | **GSE21653** | 266 | Affymetrix Human Genome U133 Plus 2.0 Array |
| **GSE2603** | 82 | Affymetrix Human Genome U133A Array |  | **GSE42568** | 104 | Affymetrix Human Genome U133 Plus 2.0 Array |
| **GSE2990** | 189 | Affymetrix Human Genome U133A Array |  | **GSE48390** | 81 | Affymetrix Human Genome U133 Plus 2.0 Array |
| **GSE3494** | 251 | Affymetrix Human Genome U133A Array |  | **GSE58812** | 107 | Affymetrix Human Genome U133 Plus 2.0 Array |
| **GSE6532** | 327 | Affymetrix Human Genome U133A Array |  | **GSE17907** | 51 | Affymetrix Human Genome U133 Plus 2.0 Array |
| **GSE7390** | 198 | Affymetrix Human Genome U133A Array |  | **GSE20711** | 88 | Affymetrix Human Genome U133 Plus 2.0 Array |
| **GSE11121** | 200 | Affymetrix Human Genome U133A Array |  | **GSE20685** | 327 | Affymetrix Human Genome U133 Plus 2.0 Array |
| **GSE12093** | 136 | Affymetrix Human Genome U133A Array |  | **GSE61304** | 58 | Affymetrix Human Genome U133 Plus 2.0 Array |
| **GSE17705** | 298 | Affymetrix Human Genome U133A Array |  | **GSE58984** | 94 | Affymetrix Human Genome U133 Plus 2.0 Array |
| **GSE25055** | 310 | Affymetrix Human Genome U133A Array |  | **GSE6532** | 87 | Affymetrix Human Genome U133 Plus 2.0 Array |
| **GSE25065** | 198 | Affymetrix Human Genome U133A Array |  | **GSE53031** | 113 | Affymetrix Human Genome U219 Array |
| **GSE25066** | 508 | Affymetrix Human Genome U133A Array |  | **GSE86166** | 366 | Human RSTA Custom Affymetrix 2.0 microarray |
| **GSE37181** | 123 | Illumina HumanWG-6 v3.0 beadchip |  |  |  |  |

Table S1. GEO datasets used in this study


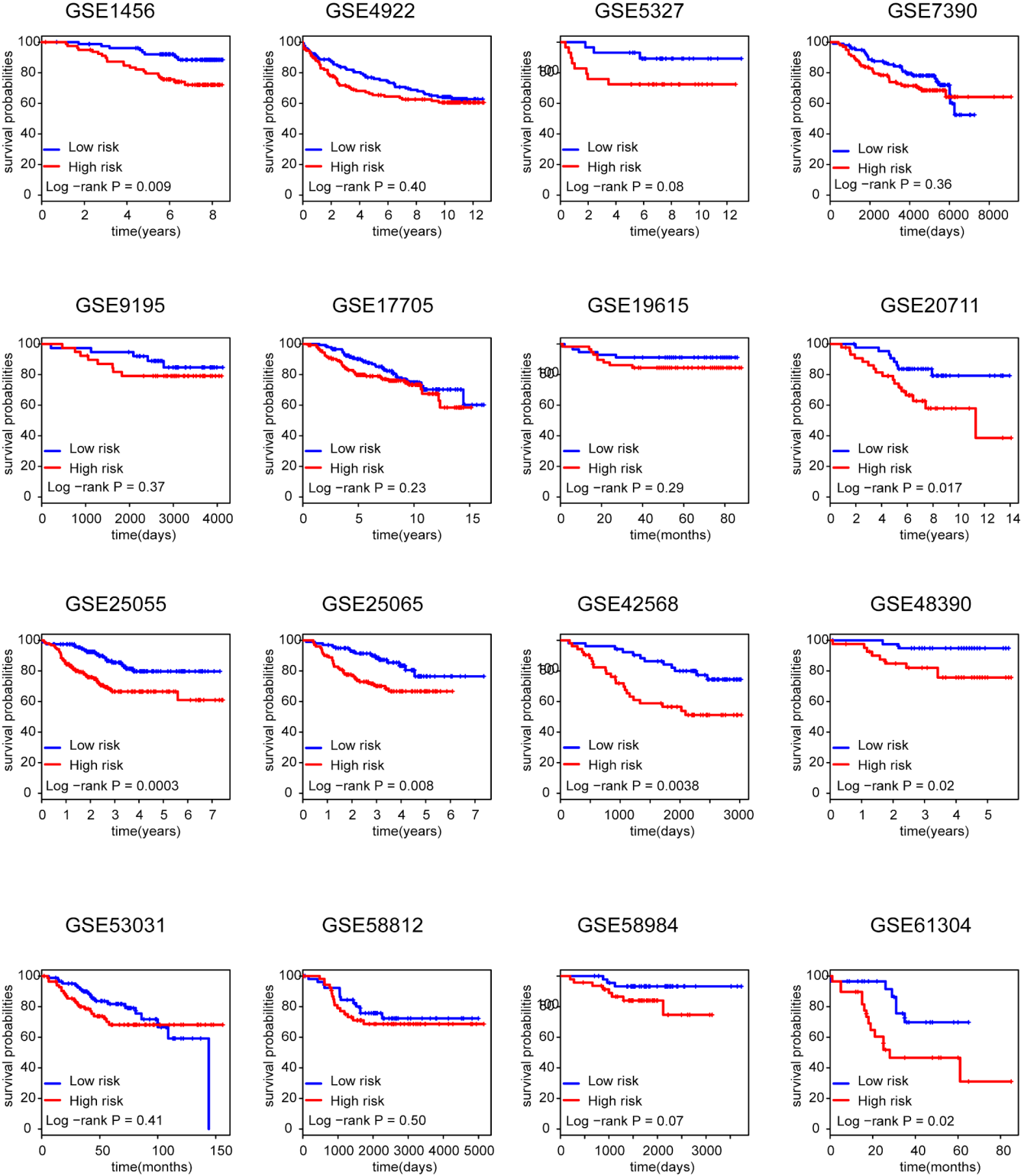


*Figure S1. Transcriptional regulator model predicts survival in breast cancer datasets.* Kaplan-Meier curves of overall survival for independent microarray data sets. The low-risk and high-risk groups of patients were determined on the basis of the median risk score for each validation dataset.


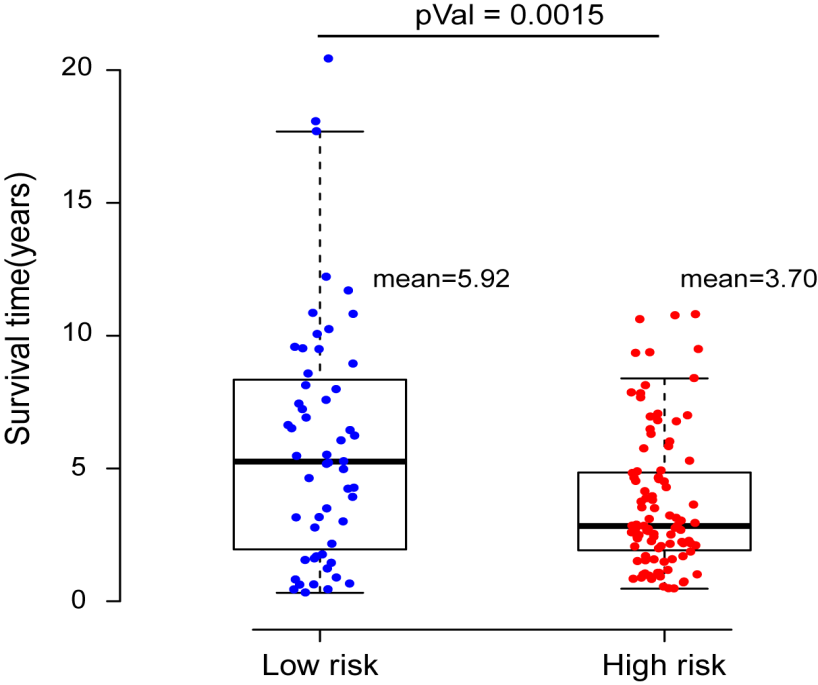


Figure S2. The regulator risk groups associated with average survival time. The high-risk group patients tend to have a shorter survival time compared with the low risk patients.


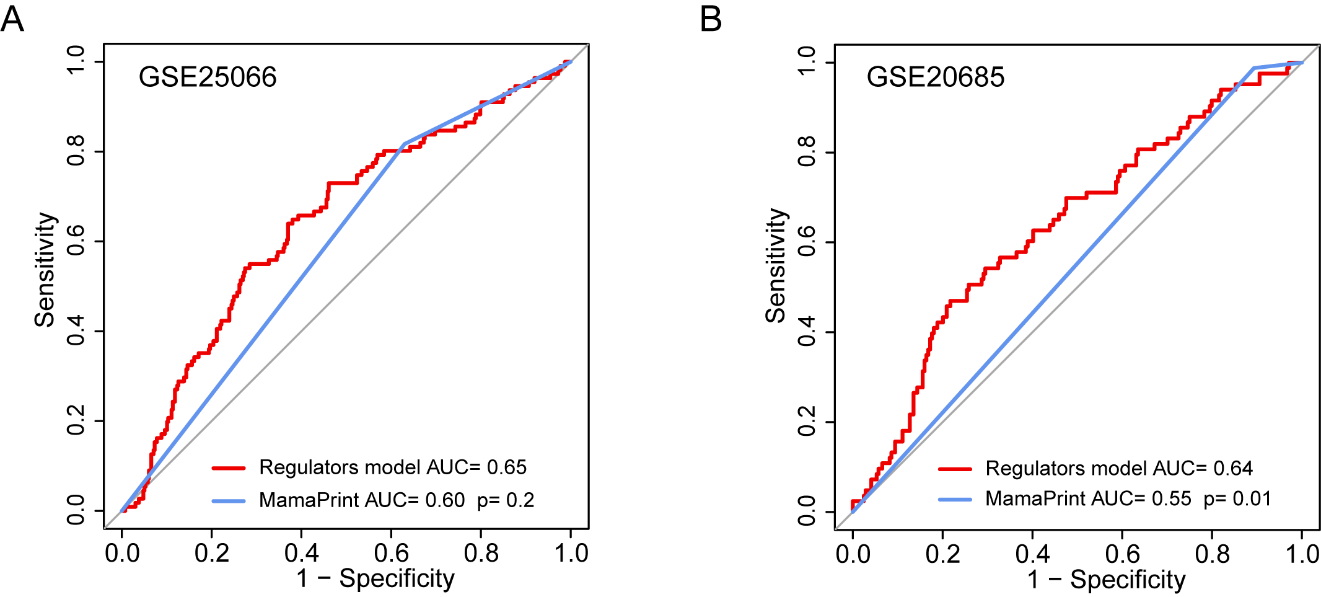


Figure S3. Comparison of the performance of the regulator model with MammaPrint. ROC curves of the regulator model and MammaPrint in predicting overall survival in two of the biggest validation sets GSE25066 (A) and GSE20685 (B).
